# Self-aligned multi-layer X-ray absorption grating using large-area fabrication methods for X-ray phase-contrast imaging

**DOI:** 10.1038/s41598-023-29580-2

**Published:** 2023-02-13

**Authors:** Abdollah Pil-Ali, Sahar Adnani, Karim S. Karim

**Affiliations:** 1 Department of Electrical and Computer Engineering, University of Waterloo, 200 University Ave W, Waterloo, ON N2L3G1 Canada; 2 Centre for Bioengineering and Biotechnology, University of Waterloo, 200 University Ave W, Waterloo, ON N2L3G1 Canada

**Keywords:** X-rays, Optical materials and structures

## Abstract

X-ray phase-contrast (XPCi) imaging methods are an emerging medical imaging approach that provide significantly better soft tissue contrast and could function as a viable extension to conventional X-ray, CT, and even some MRI. Absorption gratings play a central role in grating-based XPCi systems, especially because they enable the acquisition of three images in a single exposure: transmission, refraction, and dark-field. An impediment to commercial development and adoption of XPCi imaging systems is the lack of large area, high aspect ratio absorption gratings. Grating technology development, primarily due to technological limitations, has lagged system development and today prevents the scaling up of XPCi system into a footprint and price point acceptable to the medical market. In this work, we report on a self-aligned multi-layer grating fabrication process that can enable large-area X-ray absorption gratings with micron-scale feature sizes. We leverage large-area fabrication techniques commonly employed by the thin-film transistor (TFT) display industry. Conventional ITO-on-glass substrates are used with a patterned film of Cr/Au/Cr that serves as a self-aligned lithography mask for backside exposure. Commonly available SU-8 photoresist is patterned using the backside exposure mask followed by an electroplating step to fill the gaps in the SU-8 with X-ray attenuating material. Consequently, the electroplated patterned material acts as a self-aligned photomask for subsequent SU-8 layer patterning and so forth. The repeatability of the reported process makes it suitable for achieving higher aspect ratio structures and is advantageous over previously reported X-ray LIGA approaches. A prototype three-layer grating, with a thickness of around $40\,\upmu \text{m}$, having a visibility of 0.28 at $60\,\text{kV}_p$ with a $70\,\text{mm}\times 70\,\text{mm}$ active area was fabricated on a 4-inch glass substrate and demonstrated by modifying a commercially available 3D propagation-based XPCi Microscope. The scalable and cost-effective approach to build larger area X-ray gratings reported in this work can help expedite the commercial development and adoption of previously reported Talbot-Lau, speckle-tracking, as well as coded-aperture XPCi systems for large-area clinical and industrial applications.

## Introduction

X-ray phase-contrast is a relatively new X-ray imaging method that provides greatly improved soft-tissue visualization with X-ray making it a potential extension to conventional X-ray imaging systems as an alternative to expensive and bulky CT and MRI^1,2^. Applications that are enabled by X-ray phase-contrast imaging (XPCi) span many fields, from industry and science to medicine and biology^3–6^. Among various techniques developed to realize XPCi, Talbot-Lau^7^, speckle-tracking^8^, and coded-aperture (edge-illumination)^9^ all use X-ray absorption gratings. These techniques can provide higher contrast and sensitivity compared to propagation-based XPCi technique. Grating-based XPCi techniques can potentially provide three images for each single exposure: transmission (absorption), refraction (phase), and dark-field (small angle scattering)^10,11^. The single-exposure grating-based XPCi techniques result in less delivered dose to the sample while shortening the imaging time—compared to multi-exposure stepping or scanning methods^9,12^. The additional images—phase and scattering—help further quantify object characteristics beyond conventional attenuation X-ray imaging. X-ray absorption gratings are typically fabricated using high aspect ratio X-ray absorber structures to transform incoming X-rays into individual beamlets. Any imperfection in the fabrication process directly affects the final image quality, leading to image artifacts, inconsistencies, and/or loss of image quality and detail^13–15^.

Additional challenges for grating fabrication include higher resolution, higher energies, and larger field-of-view (FOV). For example, high-resolution X-ray detectors are necessitated by some clinical, material science, and industrial inspection applications^15–20^. Higher resolution applications require periodic gratings with a high aspect ratio for structures in the micron-scale range to absorb X-ray photons and create fine beamlets. Furthermore, applications requiring higher X-ray energies require even higher aspect ratios and critically larger FOV—the FOV in most diagnostic medical and security applications is large, usually exceeding 30 cm$\times$30 cm^21–25^.

Several methods have been explored for high aspect ratio X-ray grating fabrication that can be categorized based on either etching or molding techniques. The former is usually conducted on silicon wafers through anisotropic etching techniques such as metal-assisted chemical etching^26,27^, plasma etching^28–30^, or wet etching^31,32^. Although silicon-based etching can achieve high aspect ratio structures, fabrication is limited to standard wafer sizes^29,33^. While tiling and stitching smaller gratings to achieve a larger grating is an option^34,35^, it is challenging to overcome tiling gaps for emerging high-resolution applications enabled by new micron-scale pixel pitch X-ray detectors^15,20^.

A widely used method for fabricating high aspect ratio microstructures is the LIGA process—lithographie, galvanoformung, abformung or alternately lithography, electroplating, and molding^36–38^. Lithography methods used in the LIGA process are either based on UV or X-ray. Although the X-ray LIGA process results in high aspect ratio structures^39^, the method requires specialized X-ray sources with highly specific beam characteristics. Moreover, fabrication of large-area gratings in X-ray LIGA is challenging due to the limited size of the X-ray beam—typical device area fabricated in X-ray LIGA is less than4-inch square^40,41^. On the other hand, conventional UV-LIGA^42^ is unable to obtain the high aspect ratios of X-ray LIGA because of the drawbacks of UV lithography, i.e., the diffraction of UV light as it travels away from the photomask into the resist. However, UV LIGA is a well-established, mature, process where large-area scaling challenges are well-understood and already resolved in the industry, such as fabrication of liquid crystal display (LCD) thin-film transistor (TFT). LCD-TFT technology can be scaled in size—in UV LIGA—as evidenced by large area flat panel television sets. While wafer scale gratings are limited to standard wafer sizes, LCD-TFTs are fabricated on meter-sized glass substrates and are already widely used for large area diagnostic X-ray imaging. UV lithography lamps can uniformly expose areas approaching 1 m $\times$ 1 m using industry-standard step and repeat processes for large area display manufacturing^43,44^. Moreover, such an approach can also piggyback on ongoing advances in large-area electronics fabrication technology including emerging flexible substrate fabrication processes and improved sensor integration.

LIGA hinges upon creating resist molds—such as with SU-8—and conducting electroplating to form the absorption part of X-ray absorption gratings. SU-8 is a negative epoxy resist^45^ that has been employed to fabricate numerous micro-electromechanical systems (MEMS) such as optical devices, micro-fluidic channels, lab-on-chip devices, and passivationlayers^46–49^. Moreover, SU-8 films exceeding 1 mm thickness have been patterned using UV exposures^50–52^ in UV LIGA. One of the well-known properties of SU-8, which makes it a suitable candidate for 3-D processing in MEMS, is its multi-layer coating compatibility^53–56^. SU-8 has been widely used in both X-ray and UV-LIGA techniques for X-ray absorption grating fabrication, but its multi-layer capability has never been reported for grating fabrication.

This research explores an alternative to tiling silicon wafers to obtain a large area X-ray grating by leveraging widespread LCD-TFT fabrication technology^57,58^. The process we report uses both SU-8 and the UV-LIGA process to stack multiple layers of lower aspect ratio grating structures to achieve a high-resolution and potentially a higher aspect ratio structure than reported to date with standard UV-LIGA techniques. UV lithography is widely available and cost-effective, and using the multi-layer approach described in this work to overcome its shortcomings is the primary technique used to achieve large-area, high-resolution gratings.

## Materials and methods

To achieve alignment accuracy between the multiple grating structure layers, we employed a UV self-alignment step through the backside of the UV transparent substrate^59^. Self-aligned fabrication processes have been reported for thin film semiconductor devices^60–62^. This technique is also used in X-ray LIGA as substrates employed in grating fabrication—usually silicon or graphite—are transparent to X-rays used for lithography. Self-aligned X-ray lithography has also been employed to fabricate double-sided X-ray absorption gratings—with a single grating layer on each side of a substrate^63,64^. However, the double-sided X-ray grating fabrication method is limited in the highest achievable aspect ratio because this method does not consider a multi-layer approach and is constrained to building the grating on only the two sides of the substrate. To the best of our knowledge, a self-aligned UV LIGA process has never been reported for multi-layer X-ray grating fabrication to date.

**Figure 1:**
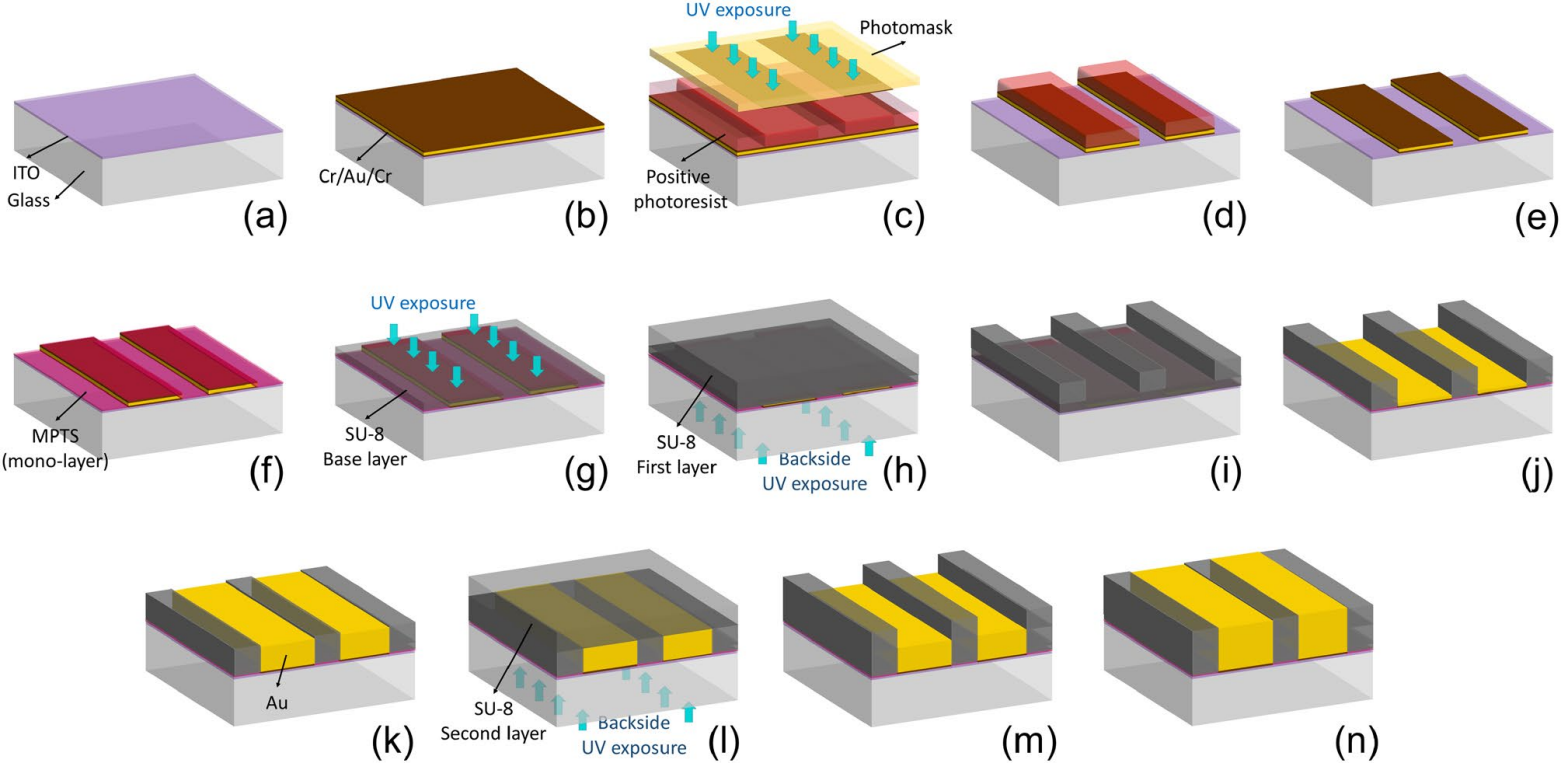
Process flow for fabricating self-aligned multi-layer high aspect ratio gratings. This graph illustrates the fabrication steps for two layers, where repeating steps (l) to (n) results in a multi-layer structure: (**a**) cleaning the ITO-on-glass substrate, (**b**) thermal deposition of Cr/Au/Cr thin films, (**c**) spin coating a positive photoresist and patterning it through a photomask, (**d**) etching uncovered Cr/Au/Cr parts, (**e**) cleaning the sample and removing the photoresist,(f) definition of a mono-layer 3-(Trimethoxysilyl)propyl methacrylate (MPTS) film, (**g**) definition of the SU-8 base layer and performing a flood exposure from the top, (**h**) spin coating the first SU-8 layer and performing a backside exposure, (**i**) developing the uncrosslinked SU-8, (**j**) performing an RIE to remove base SU-8 layer and etching the top Cr film, (**k**) gold electroplating to fill the gaps, (**l**) spin coating the second SU-8 layer and performing a backside exposure, (**m**) developing the uncrosslinked SU-8 parts, (**n**) gold electroplating to fill the gaps and so on.

### Self-aligned multi-layer fabrication process

The process is schematically illustrated in Fig. 1. First, a chromium/gold/chromium (Cr/Au/Cr) film stack (5 nm/100 nm/ 5nm) was deposited on an ITO-on-glass substrate using a thermal evaporation process with an Intlvac Thermal Evaporator (Intlvac Inc., CA) at room temperature without breaking the vacuum. The Cr/Au/Cr film was patterned on the ITO-on-glass substrate using a selective wet etching process. The chromium thin films were removed through the wet etching process at room temperature using a chromium etchant made of ceric ammonium nitrate ($(NH_4)_2[Ce(NO_3)_6]$), acetic acid ($CH_3COOH$), and deionized (DI) water. Also, gold wet etching was performed using a commercially available GE-8148 Gold Etchant (from Transene Company Inc., USA). To improve the adhesion quality of SU-8 to the gold thin film, we used a combination of a thin film of chromium (the top Cr film), a mono-layer of 3-(Trimethoxysilyl)propyl methacrylate or MPTS (from Sigma-Aldrich, USA), and a thin layer of SU-8. The mono-layer of MPTS on chromium thin film was formed through a liquid treatment step^65^—using ethanol and acetic acid—prior to the definition of a thin layer of SU-8. We made a thin layer of SU-8 by diluting SU-8 2015 with Cyclopentanone (from Sigma-Aldrich, USA)—known as SU-8 thinner—to achieve a desired thin SU-8 (referred to as the SU-8 base layer hereafter).

A 500 nm thin SU-8 base layer was spin coated followed by a soft baking step at $65\,^{\circ }\text{C}$ for 60 min via a ramping up (from room temperature) and ramping down (to room temperature) processes at a rate of $120\,^{\circ }\text{C}$ per hour, all on a hot plate (from Torrey Pines Scientific, USA). This SU-8 base layer was then flood exposed in an i-line (365 nm) using a mask aligner (Karl Suss MA6, Germany), and a post-exposure bake was performed at $65\,^{\circ }\text{C}$ for 60 min under the same conditions as the soft bake step. A hard bake step was performed for 30 min at $170\,^{\circ }\text{C}$—with the same ramping condition as the soft bake—to further improve the mechanical stability of the SU-8 base layer.

The first SU-8 layer was then exposed to a backside UV flood exposure, with the patterned Cr/Au/Cr layer acting as a photomask underneath the first SU-8 layer. After conducting the post-exposure bake, the un-crosslinked parts of the SU-8 resist were developed in either propylene glycol methyl ether acetate (PGMEA) (Sigma-Aldrich, USA) or 1-Methoxy-2-Propanol Acetate (Kayaku Advanced Materials, USA), followed by a hard bake step to ensure the mechanical stability of the final structure. Before performing metal electroplating and forming the absorption part of the grating, the SU-8 base layer was etched down to the top of the chromium thin film by an RIE dry etching process (Phantom II RIE system, Trion Technology Inc., USA) using a combination of $O_{2}$ and $CF_{4}$ gases^66^. The chromium thin film was then removed through a wet etching process at room temperature using the chromium etchant.

The sample was electroplated with metal (Au) to form the absorption part of the grating by filling the gaps between the patterned SU-8. The electroplated region thereafter acted as a self-aligned photomask for the next SU-8 layer and so on. Since the grid pattern is transferred via a self-aligned step, any offset between the self-aligned photomask and the next SU-8 layer is minimized. This fabrication process ensures a quality sidewall in SU-8 lithography and could, in principle, be repeated multiple times until the desired thickness of an absorption grating is achieved.

### Effect of exposure energy

**Figure 2:**
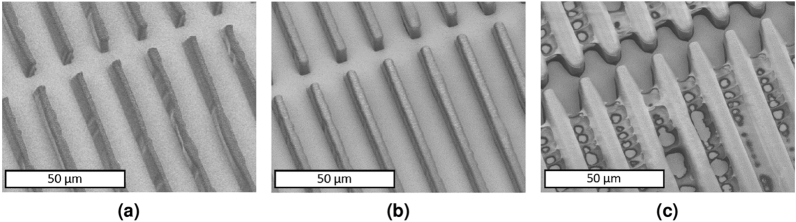
SEM images of the effect of exposure dose on the quality of pattern transfer in the self-aligned backside UV lithography. (**a**) Insufficient UV exposure dose results in unstable SU-8 features and incomplete pattern transfer. (**b**) An optimized UV exposure dose transfers the patterns onto the SU-8 layer successfully. (**c**) Higher exposure dose leads to resolution loss in features and SU-8 cross-linking in unwanted areas.

**Figure 3:**
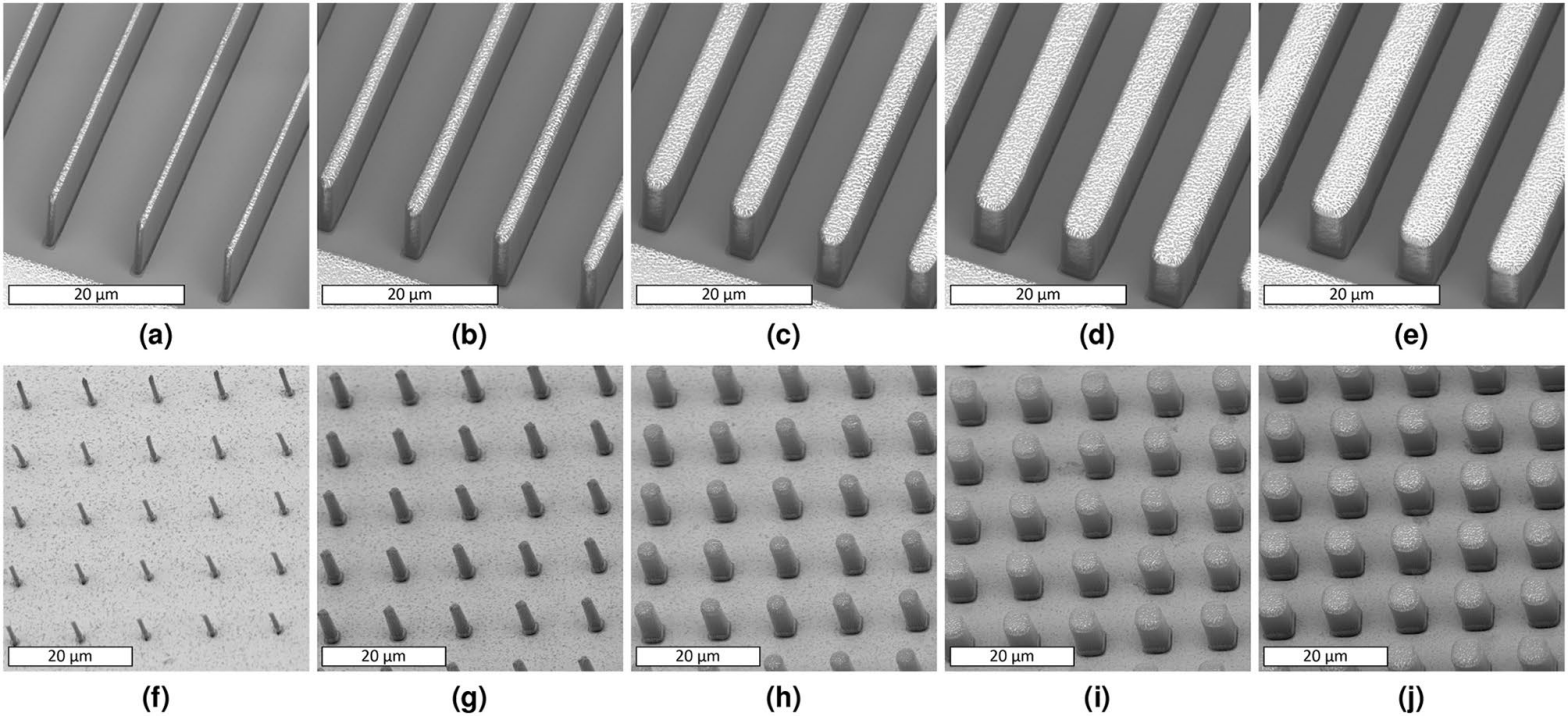
SEM images of pattern transfer of various feature sizes through the self-aligned backside UV lithography. Periodic SU-8 lines with widths of (**a**) $1\,\upmu \text{m}$, (**b**) $2\,\upmu \text{m}$, (**c**) $3\,\upmu \text{m}$, (**d**) $4\,\upmu \text{m}$, (**e**) $5\,\upmu \text{m}$; and periodic SU-8 micropillars with diameters of (**f**) $1\,\upmu \text{m}$, (**g**) $2\,\upmu \text{m}$, (**h**) $3\,\upmu \text{m}$, (**i**) $4\,\upmu \text{m}$, (**j**) $5\,\upmu \text{m}$.

The quality of the transferred pattern was found to be energy-dependent^67,68^. A successful pattern transfer in backside UV exposure lithography was achieved through an optimized UV exposure dose (energy). Experimental results of exposure dose characterization (Fig. 2) demonstrated that a low exposure dose leads to mechanically unstable SU-8 structures, incomplete pattern transfer, and incomplete cross-linking (Fig. 2a). On the other hand, a high dose exposure results in cross-linking in unexposed areas between structures and loss of resolution (Fig. 2c). SU-8 lines and micropillars feature with widths and diameters of 1–$5\,\upmu \text{m}$ (Fig. 3) were patterned in the first layer, with 10 $\upmu \text{m}$ thick SU-8, following an optimal exposure dose. The optimal exposure dose was selected based on the pattern transfer quality, feature sizes, periodicity of features, and SU-8 thickness at the dose that SU-8 features become sufficiently cross-linked with an acceptable sidewall quality.

### Electroplating

Electroplating of gold was conducted using the Elevate Gold 7990 (from Technic Inc., USA), a sodium gold sulfite solution with 8.2 g/L concentration and a slightly acidic pH (6.3). The electroplating solution was warmed up and kept at $49\,^{\circ }\text{C}$ ($121\,^{\circ }\text{F}$) using a PLC-controlled electric immersion heater, and the solution was filtered and stirred throughout the process to ensure a better electroplating uniformity across the sample. We used a platinized titanium mesh anode with a surface area greater than our samples, which was kept 10 cm away from the sample. Electroplating was performed at 3 $\text{mA}/\text{cm}^2$ using a constant current source, resulting in a growth rate of around 100 nm/min. Samples were cleaned and washed using de-ionized (DI) water both before immersion into the electroplating solution and after the electroplating was completed. Samples were dried with nitrogen ($\text{N}_2$) after being rinsed at the end of electroplating.

**Figure 4:**
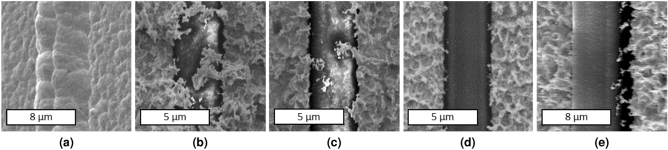
SEM images of gold electroplating. (**a**) An overplated sample where SU-8 features are buried under excessive gold plated. (**b**) Sample has undergone a short gold wet etching process. (**c**) A longer gold wet etching starts opening the SU-8 features. (**d**) SU-8 features are open and excessive plated gold is completely etched. (**e**) A side view of released Su-8 features.

High-quality electroplating for successful self-aligned lithography in our design required that samples not be over-plated. Over-plating results in the UV transparent regions on the sample becoming covered and obscured, warping the shape of the grid in subsequent layers. To avoid such adverse behavior (i.e., over-plating effects), samples with over-plated gold were partially wet etched using the gold etchant. Gold etchant was diluted in DI water (1:2 vol/vol) to have better control over the etching rate. Fig. 4 illustrates SEM images of a SU-8 line buried under over-plated gold (Fig. 4a), which had undergone a partially gold wet etching process (Fig. 4b, c) until the excessive gold electroplated regions were removed, and the SU-8 line was released (Fig. 4d). Fig. 4a, e are SEM images taken at an angle to demonstrate a better view of over-plated gold and the etched gold, respectively.

**Figure 5:**
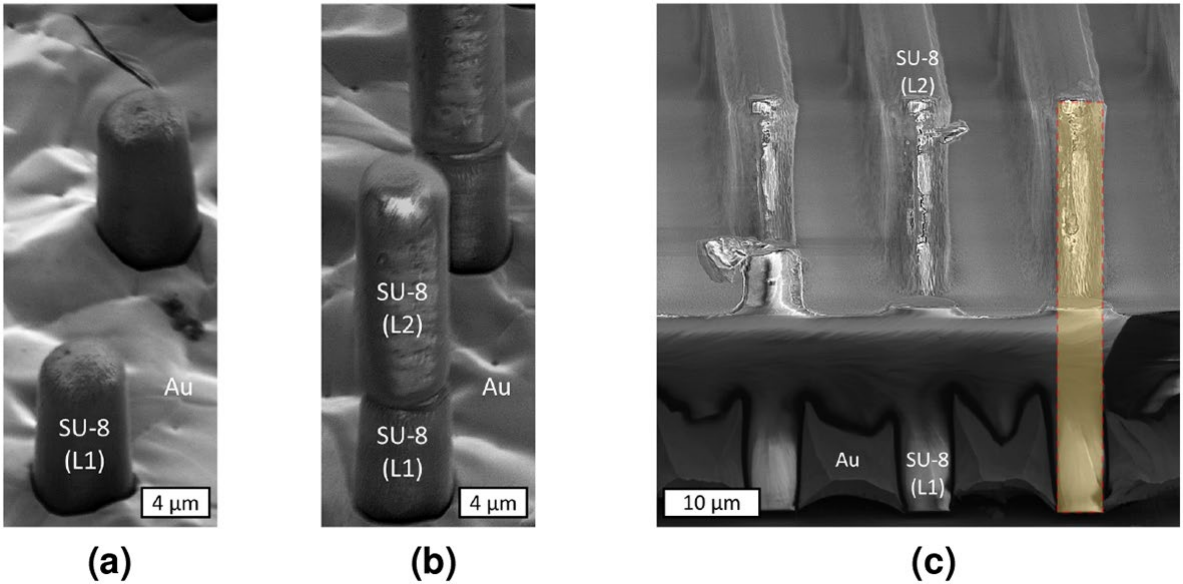
SEM images of SU-8 micropillar and line gratings through the self-aligned multi-layer fabrication process. (**a**) Demonstration of SU-8 micropillars before the definition of the second SU-8 layer, and (**b**) the same SU-8 micropillars after the second SU-8 layer (micropillars) is fabricated following the self-aligned backside UV lithography. (**c**) Illustration of a two-layer SU-8 lamella structure—for line grating.

### Pattern transfer to subsequent layer

After successfully electroplating and forming the absorption grating, samples were rinsed with DI water, dried with $\text{N}_2$, and the subsequent SU-8 layer was coated to make the next grid layer. Subsequent self-aligned backside UV exposures required higher exposure energy to compensate for the energy loss in the previous SU-8 layers. Hard-baked SU-8 indicates almost 10$\%$ loss in the UV transmission at 405 nm^69^, the wavelength of our mask aligner, which could simply be compensated by increasing exposure time by 10$\%$. After developing samples in the SU-8 developer, a hard bake step was also carried out to enhance the mechanical stability of SU-8 structures. Figure 5 illustrates SEM images of micropillars before (5a) and after (5b) patterning the second SU-8 layer through the self-aligned backside UV exposure. While SU-8 micropillars in Fig. 5a were underplated, they resulted in an acceptable pattern transfer from the first SU-8 layer. The same scenario, an underplated first SU-8 layer, was observed on line gratings, where a complete pattern transfer of structures was achieved through the self-aligned backside UV exposure technique. Figure 5c depicts an SEM image of a SU-8 line grating with two SU-8 layers (the image was taken before the unexposed region of the second SU-8 layer was developed entirely).

**Figure 6:**
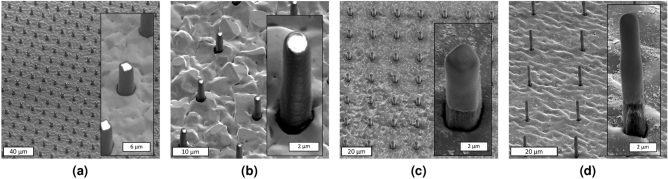
SEM images of the second SU-8 layer in various feature sizes of SU-8 micropillar-based gratings. A successful pattern transfer from the first SU-8 layer to the second one is evident in all samples, with (**a, b**) being the SU-8 micropillars sufficiently electroplated, and (**c, d**) being the SU-8 micropillars underplated.

Increasing the number of layers resulted in structures with a high aspect ratio, which was practiced to fabricate the prototype version of a large-area X-ray grating. SEM images of various feature sizes of the second SU-8 layer are illustrated in Fig. 6. We investigated the quality of the second SU-8 layer backside UV exposure on a variety of samples based on the quality of first layer electroplating. In Fig. 6a, b SU-8 micropillars in the first SU-8 layer were sufficiently electroplated. In Fig. 6c, d, on the other hand, structures in the first SU-8 layer were slightly underplated. Figure 6d illustrates SU-8 micropillars fabricated in a two-layer structure with diameters of 1.5 $\upmu \text{m}$ (Fig. 6d), an aspect ratio of around 13:1 (height to diameter ratio of SU-8 micropillars), and a total height of around 20 $\upmu \text{m}$ with two layers of SU-8—the second layer electroplating is not conducted.

**Figure 7:**
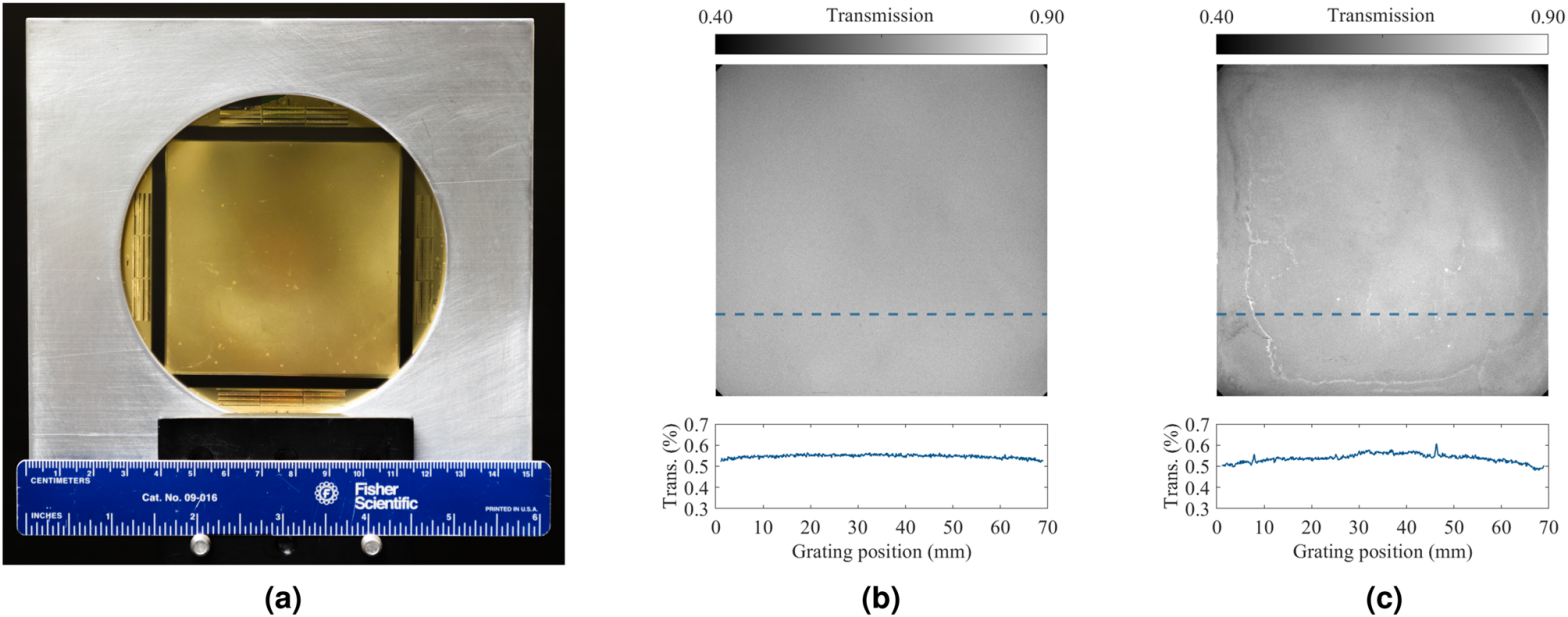
(**a**) Demonstration of a prototype large-area $70\,\text{mm}\times 70\,\text{mm}$ grating fabricated on a 4-inch glass substrate. Transmission map at $60\,\text{kV}_p$ of (**b**) a two-layer and (**c**) a three-layer 70 mm $\times$ 70 mm prototype X-ray absorption grating. The three-layer grating exhibits a few visible blemishes stemming from the third-layer electroplating.

### Large-area grating fabrication

To demonstrate the self-aligned multi-layer fabrication process, a prototype three-layer SU-8 micropillar-based (2D) grating with 4 $\upmu \text{m}$ aperture size and 16.2 $\upmu \text{m}$ period was fabricated with dimensions of $70\,\text{mm}\times 70\,\text{mm}$ on a 4-inch glass substrate of $700\,\upmu \text{m}$ thickness. The final electroplated gold thickness for the prototype three-layer X-ray grating was around $40\,\upmu \text{m}$—an aspect ratio of around 10:1. Figure 7a demonstrates the $70\,\text{mm}\times 70\,\text{mm}$ prototype grating mounted on an aluminum holder for easier handling. The transmission image at $60\,\text{kV}_p$ of a two-layer and a three-layer version of the $70\,\text{mm}\times 70\,\text{mm}$ prototype grating is presented in Fig. 7b, c, respectively, along with a cross-sectional profile across the dashed blue line in each image representing the corresponding grating transmission value and its uniformity. Table 1 summarizes the specifications of the two-layer and the three-layer version of the $70\,\text{mm}\times 70\,\text{mm}$ prototype gratings.

### X-ray phase-contrast imaging setup

**Figure 8:**
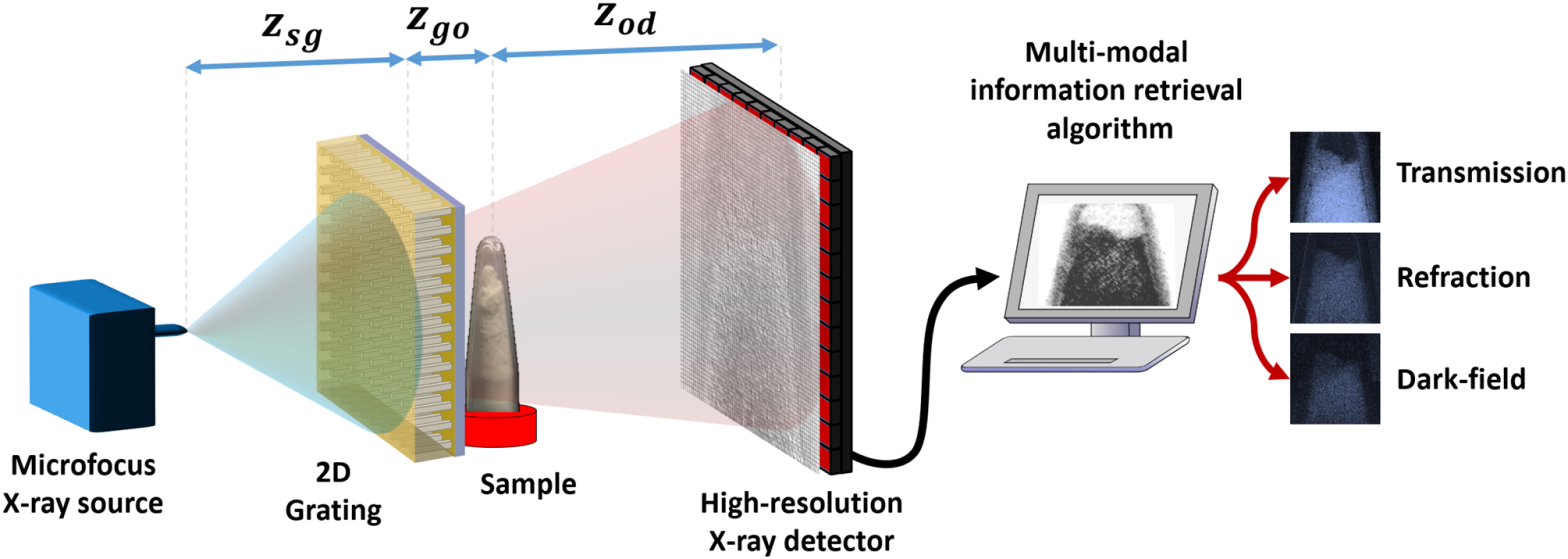
Schematic diagram of the X-ray setup used to perform X-ray phase-contrast imaging using a single X-ray absorption grating.

X-ray phase-contrast imaging was performed using a commercially available in-line phase-contrast 3D X-ray Microscope—inCiTe™ (from KA Imaging Inc., Waterloo, CA)—which utilizes a polychromatic microfocus X-ray source—Hamamatsu X-ray source L12531 (from Hamamatsu Photonics, Shizuoka, JP) with a minimum focal spot size of $2\,\upmu \text{m}$—along with an early prototype 4K $\times$ 4K high-resolution direct conversion X-ray detector with $8\,\upmu \text{m}$ pixel size^15,20^. Figure 8 demonstrates a schematic diagram of the X-ray setup used in this work.

**Table 1:** Specifications of the prototype two-layer and three-layer gratings.

Grating	Spec.					
	Number of SU-8 layers	Number of electroplated layers	Aspect ratio of SU-8 features (height:width ratio of resist)	Absorption layer total thickness ($\upmu \text{m}$)	Diameter of micropillars ($\upmu \text{m}$)	Periodicity of micropillars ($\upmu \text{m}$)
2-Layer	2	2	7:1	$\approx 35$	5	27
3-Layer	3	3	10:1	$\approx 40$	4	16.2

## Results and discussion

### High-resolution X-ray transmission image

**Figure 9:**
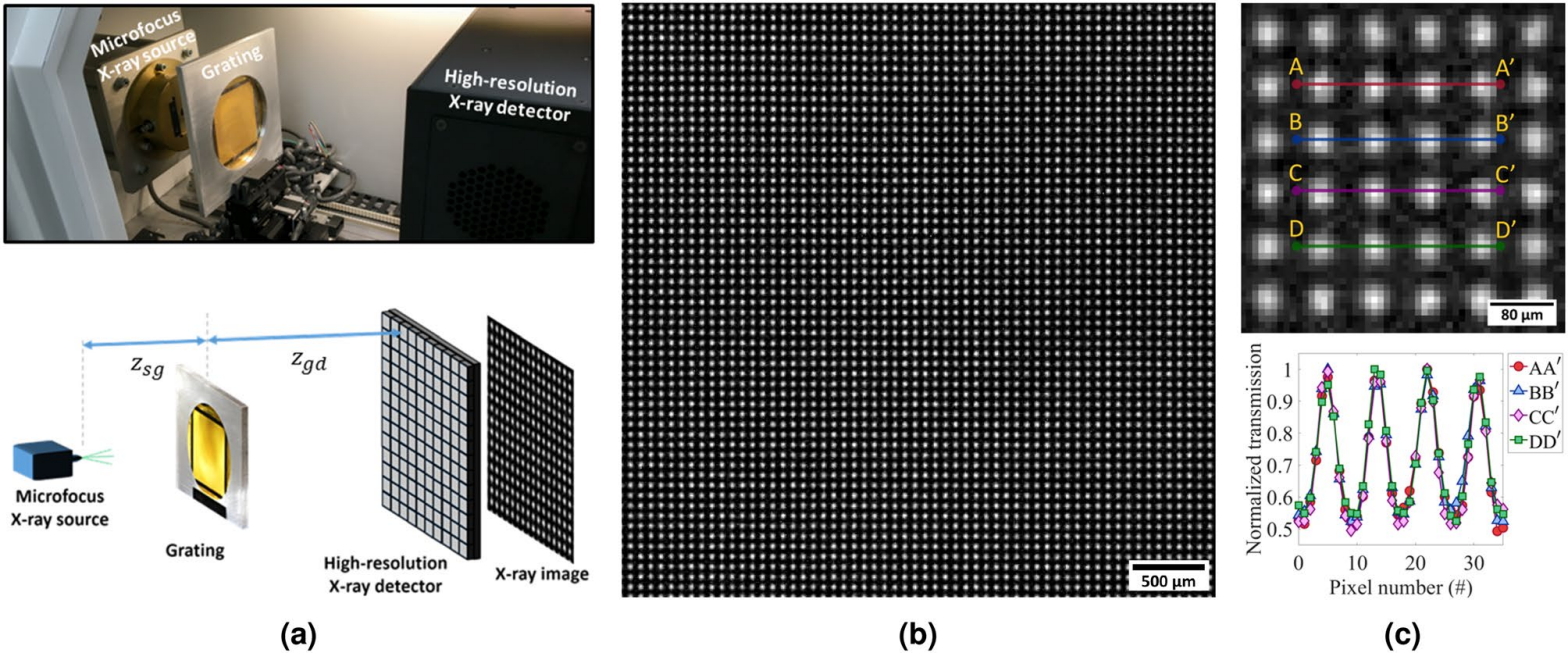
(**a**) Demonstration of the 3D X-ray Microscope—inCiTe™—and the test setup we used for taking X-ray projection of the prototype version of our grating. (**b**) X-ray projection of the SU-8 micropillar-based X-ray grating with three layers at $60\,\text{kV}_p$; a magnification of 4.5 was used in this X-ray projection. (**c**) A closer view of grids in the X-ray projection image (top), and the cross-sectional profiles of normalized transmission of grids across four rows of grids (bottom).

A final inspection of the prototype X-ray absorption grating was carried out—employing the setup depicted in Fig. 9a—by measuring the X-ray transmission of the fabricated three-layer SU-8 micropillar-based grating. We took an X-ray image of the fabricated grating—as illustrated in Fig. 9b—with the X-ray source at 60 $\text{kV}_p$ and 200 $\upmu$A, source to grating distance ($\text{z}_{{sg}}$) of 10 cm and grating to detector distance ($\text{z}_{{gd}}$) of 35 cm. These distances were selected to have a magnified image of grids on the detector so that their profile could be resolved. A closer view of the grids profiles and cross-sectional graphs of their normalized transmission profiles are shown in Fig. 9c. As evident in the transmission image in Fig. 9c, the SU-8 micropillar structures let the X-ray go through, and the electroplated gold region in between the SU-8 micropillars absorbs the X-rays.

**Figure 10:**
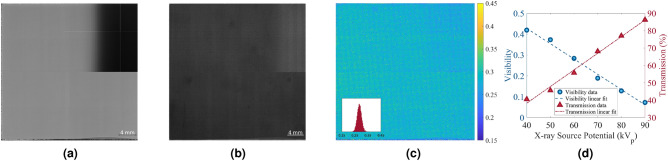
(**a**) Gain map of the prototype 4K $\times$ 4K high-resolution X-ray detector, showing detector defects and artifacts; the top right quarter of the detector is defective. (**b**) Transmission image for part of the prototype fabricated grating at $60\,\text{kV}_p$. (**c**) Visibility map for the prototype fabricated grating. (**d**) Visibility and transmission profiles versus X-ray source potential.

### Visibility map

A visibility map of the prototype grating was also generated using the prototype 4K $\times$ 4K high-resolution X-ray detector with the X-ray source working at 60 $\text{kV}_p$. There are some pixel defects associated with the prototype X-ray detector that are visible even after dark offset and gain corrections. Figure 10a shows the gain map of the detector, which demonstrates the detector defects and artifacts. Figure 10b illustrates the transmission image for part of the prototype X-ray grating fabricated in this work. Whereas some detector artifacts are corrected after gain (and offset) correction, the detector defective areas and pixel clusters remain in the X-ray projection. The visibility map of the same part of the prototype grating employed for XPCi in this work is illustrated in Fig. 10c. The visibility map was calculated considering the maximum and minimum values of individual micropillar X-ray projections in the transmission image. Visibility and transmission characteristics of the fabricated grating were investigated at various X-ray energies shown in Fig. 10d. Although the results in Fig. 10d are sufficient for XPCi, higher visibility, if desired, could be achieved by increasing the number of layers in the multi-layer grating fabrication process to create a higher contrast between the absorbed and transmitted X-ray patterns on the X-ray projection.

### X-ray phase-contrast imaging results

**Figure 11:**
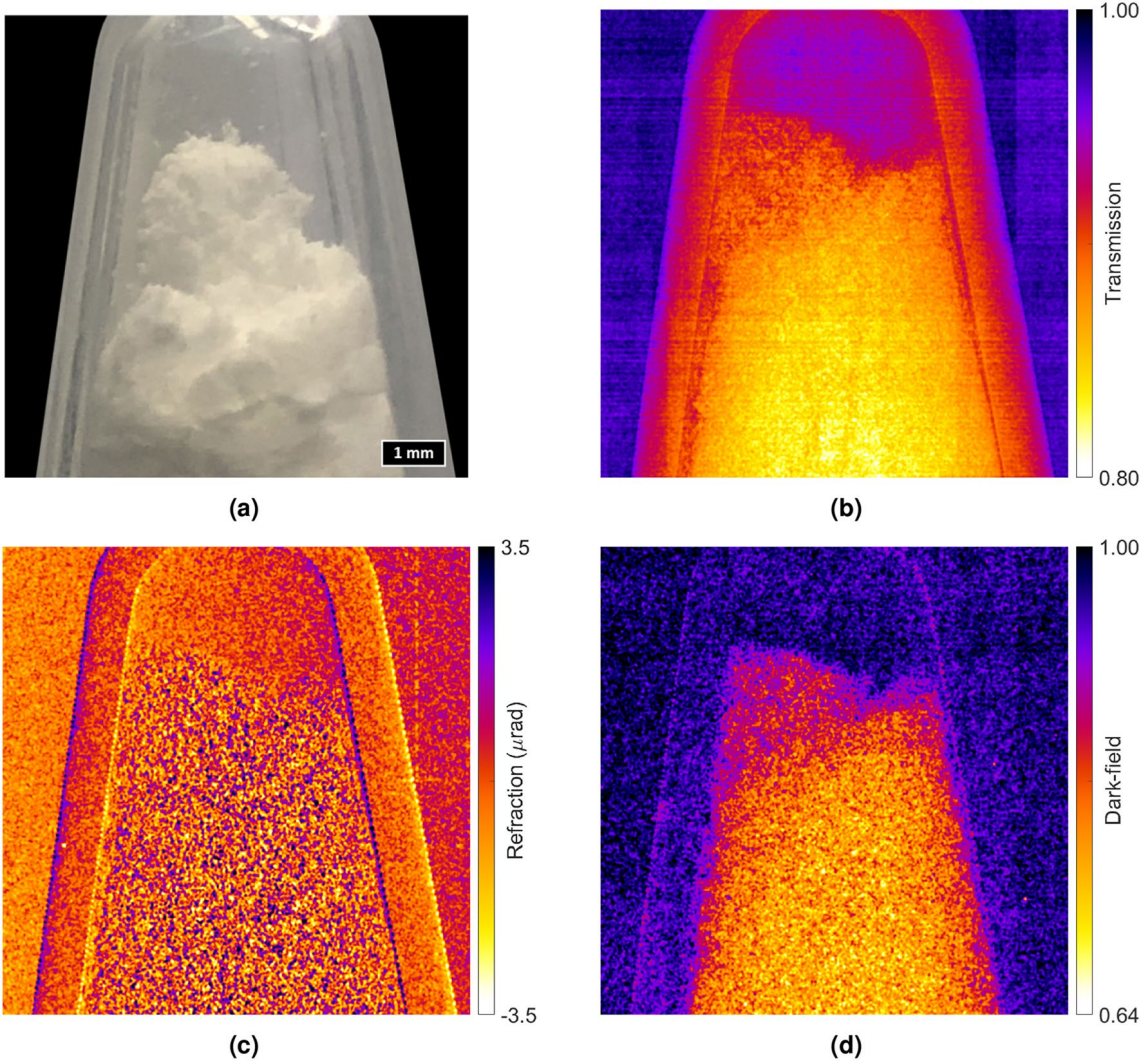
(**a**) A centrifuge tube filled with sugar powder as the sample of interest. (**b**) Transmission, (**c**) refraction, and (**d**) dark-field images retrieved from a single-exposure X-ray phase-contrast image taken at 60 $\text{kV}_p$ of the sample—images are represented based on a false color tone for better representation. As evident in the three contrast images, the grating artifacts do not show up in the final processed images.

The commercially available in-line XPCi system, inCiTe™, was modified using the fabricated SU-8 micropillar grating as the pre-sample mask of a single-mask XPCi system design. A centrifuge tube filled with sugar powder was used for the imaging task, as shown in Fig. 11a. The microfocus X-ray source was set to at $60\,\text{kV}_p$ and $200\,\upmu \text{A}$ with 40 s exposure. The source to grating distance ($\text{z}_{{sg}}$), the grating to object distance ($\text{z}_{{go}}$), and the object to detector distance ($\text{z}_{{od}}$) were set to 10 cm, 2 cm, and 33 cm, respectively. Imaging was performed in single-exposure mode, and two separate images—reference image and sample image—were captured. The reference image was obtained without the object of interest present in the imaging setup, and the sample image was taken when the object was introduced to the system. We employed unified modulated pattern analysis (UMPA)^70^ with a window size of 19 pixels to retrieve multi-modal information, including transmission, refraction (phase), and dark-field (small-angle scattering) simultaneously. The transmission, refraction, and dark-field images obtained from the scan are shown in Fig. 11b–d, respectively.

The fabricated SU-8 micropillar grating enabled the acquisition of additional refraction and dark-field image information in a single X-ray exposure using the existing geometry of a commercial in-line X-ray phase-contrast micro-CT. Although the reported results in Fig. 11 do not offer a high signal-to-noise ratio, they are especially significant in light of the ultra-compact geometry of the XPCi system due in large part to the $8\,\upmu \text{m}$ pixel pitch prototype 4K $\times$ 4K high-resolution direct conversion X-ray detector and the prototype grating reported in this work. The reported result, due to a single exposure scan, also demonstrates promise for the reduction of the delivered dose to the sample, which could be potentially beneficial for biological samples and in vivo imaging applications. Further system-level optimizations for dose could further result in lowering of the sample radiation.

### Discussion

It should be noted that silicon wafers cannot be employed readily as a substrate in the reported UV-LIGA self-aligned multi-layer fabrication process since a self-aligned design requires UV transparent substrates (e.g., glass). Moreover, the glass substrates require ITO (or another UV transparent conductive coating), because, depending on the grating design, the patterned Cr/Au/Cr film could result in discrete areas that must be electrically connected for the electroplating process to work correctly. The ITO film—a UV transparent conductive layer—ensures an electrical connection between the discrete areas of the electroplating seed layer even if the Cr/Au/Cr film has electrically disconnected islands.

Adhesion of SU-8, particularly the 2000 series, to gold thin film is not strong^71,72^, which causes the structural integrity of SU-8 molds to be compromised unless an adhesion layer—or a combination of multiple adhesion layers—is employed. It has been previously reported that Cr/MPTS combination improves the adhesion of SU-8 to gold thin film^71^, however, we also utilized a thin layer of SU-8 to further improve the adhesion of subsequent thick SU-8 structural layers to the substrate. The top chromium layer (in Cr/Au/Cr film) not only improved the adhesion of the SU-8 structural layer to the gold film, but also protected the gold film from being attacked by the dry etching gases and contaminating the RIE chamber. Our experimental results showed that the combination of a thin layer of chromium, a mono-layer of MPTS, and a thin layer of hard-baked SU-8 guaranteed that the subsequent thicker SU-8 layers did not delaminate, peel off, or break.

The thickness of SU-8 layers depends on the minimum feature size of lamellae (for line gratings) or micropillars (for 2D gratings) being fabricated in the corresponding layer, thus an acceptable sidewall quality and pattern transfer can be achieved. The maximum achievable aspect ratio in the conventional UV LIGA process is the limitation for each layer in the reported self-aligned multi-layer process. For the proof-of-concept and the prototype version of the grating—fabricated and reported in this work—we used SU-8 2015 (thickness of $12{-}16\,\upmu \text{m}$) and kept the aspect ratio of features below 4 for each layer—an unoptimized process. Further optimizations will result in a higher aspect ratio for each layer.

With regards to common defects observed in the grating, the most prominent one occurs when the grating is over- or under-exposed, leading to a change in feature size. Another prominent defect is due to the quality of the electroplating, which can also lead to a change in feature size. While the prototype two-layer grating, illustrated in Fig. 7b, has no easily distinguished blemishes, the prototype three-layer grating in Fig. 7c exhibits a few visible artifacts. The artifacts are representative of deterministic non-uniformities stemming from the grating fabrication process (primarily, the third electroplating step in the prototype grating of this work) and, being deterministic, are correctable as noted previously in Fig. 11.

The present work is the first demonstration of the multi-layer fabrication process undertaken in a university lab with no specialized equipment. Even with our first prototype, we achieved a total absorption grating thickness of $40\,\upmu \text{m}$ for SU-8 micropillars of $4\,\upmu \text{m}$ in diameter (with three layers of SU-8 resulting in structures with an aspect ratio of 10:1), and demonstrated aspect ratios of up to 13:1 with $1.5\,\upmu \text{m}$ diameter SU-8 micropillars (with two layers of SU-8), all following an unoptimized process using SU-8 2015 which provided only a thickness of 12–$16\,\upmu \text{m}$ for each layer. If we consider phase contrast mammography application, where a polychromatic beam of $30\,\text{kV}_p$ (i.e., X-ray energies of around 22 keV) is typical, then a $40\,\upmu \text{m}$ thick gold grating that stops more than 90$\%$ of the incident 22 keV X-ray photons is potentially sufficient. This implies the prototype grating fabricated in this work has an X-ray attenuation profile that is suitable for 30 kVp X-ray mammography, a prominent X-ray phase contrast medical imaging application.

## Conclusion

In this work, we presented a scalable fabrication process for large-area, high-resolution X-ray absorption gratings. The reported method relies on a self-aligned multi-layer technique based on a conventional UV-LIGA lithography process to fabricate high aspect ratio micron-scale structures. Furthermore, the process does not require any specialized processing steps or equipment, including access to synchrotron facilities for X-ray LIGA, DRIE, or even ALD commonly required in silicon-based fabrication processes. The research reported in this manuscript is both novel and impactful as the self-aligned multi-layer fabrication process can be applied to a variety of gratings for both high- and low-resolution structures in either linear or 2D implementations, using commonly available UV LIGA—or even X-ray LIGA if required. In theory, if we were to apply our multi-layer technique to both sides of the silicon or graphite substrates with X-ray lithography or to both sides of glass substrates with UV lithography, we could achieve even higher aspect ratios—a double-sided multi-layer X-ray grating.

In this work, we have demonstrated a fabrication method to make either a 1D or 2D grating using a large-area compatible process. By stacking multiple layers of the same grating design (multi-layer structures) through a backside self-aligned UV flood exposure technique, larger area X-ray gratings can be realized in an established and mature large-area thin-film electronics processing facility. We demonstrated the process by building an SU-8 micropillar-shaped (2D) three-layer X-ray absorption grating with $4\,\upmu \text{m}$ aperture size and $16.2\,\upmu \text{m}$ period (thickness of around $40\,\upmu \text{m}$), with an average visibility of 0.28 at $60\,\text{kV}_p$.

X-ray absorption gratings are fundamental components in all grating-based X-ray phase-contrast imaging systems, as they directly determine all salient image characteristics. Gratings built using our reported method could be subsequently used with small or large area detectors as required by the application. The prototype grating fabricated in this work possesses sufficient thickness to absorb $30\,\text{kV}_p$ X-rays that could potentially be employed for XPCi in mammography applications. This research has addressed a key technological bottleneck in the commercial deployment of grating-based X-ray phase-contrast imaging systems and paves the way for both clinical and non-clinical applications of XPCi where better visibility, higher X-ray energies, larger field-of-view, higher spatial resolution, and compact imaging are desirable.

## Supplementary Information

Supplementary Information.

## Data Availability

All data generated or analyzed during this study are included in this manuscript and its supplementary information files. Further requests or correspondence should be addressed to A.P.
