# Supplementary material for: Self-aligned multi-layer X-ray absorption grating using large-area fabrication methods for X-ray phase-contrast imaging
Source: Sci Rep. 2023 Feb 13;13:2508. doi: 10.1038/s41598-023-29580-2 (PMC9925796; doi:10.1038/s41598-023-29580-2)
Supplement: Supplementary file 1 — Supplementary Information. [file 41598_2023_29580_MOESM1_ESM.zip › Supplementary file/Supplementary Information.docx]

**Self-Aligned Multi-Layer X-ray Absorption Grating
using Large-Area Fabrication Methods for X-ray
Phase-Contrast Imaging**

Supplementary information

The supplementary material contains two X-ray image files: “*Sample_Image*” and “*Reference_Image*.” The sample of interest was a centrifuge tube filled with sugar powder---as explained under the “X-ray phase-contrast imaging results.” Images were taken employing the reported X-ray absorption grating of the manuscript.

Both images have the following specifications:

| **Parameter** | **Value** |
| --- | --- |
| **Image Type** | 16-bit Unsigned (Little-endian byte order) |
| **Image Width** | 4096 pixels |
| **Image Height** | 4096 pixels |

X-ray phase-contrast imaging geometry and specifications can be found in the original manuscript. Following information retrieval algorithms and using the attached images, one could retrieve transmission, phase-shift (refraction), and scattering (dark-field) information.
